# PD187 Health Technology Assessment Training Program in Ukraine: An Example Of Collaborative Capacity Building

**DOI:** 10.1017/S0266462324004094

**Published:** 2025-01-07

**Authors:** Wietske Kievit, Oresta Piniazhko, Rabia Sucu, Wija Oortwijn

## Abstract

**Introduction:**

In 2017 health technology assessment (HTA) was included in the Ukrainian Health Law fundamentals, and its implementation has accelerated as it became mandatory by 2020. As part of the Safe, Affordable, and Effective Medicines for Ukrainians project, funded by the U.S. Agency for International Development (USAID), to support the Ministry of Health in Ukraine, we developed, conducted, and evaluated a need-based HTA training program for doers, users, and trainers from July 2022 to August 2023.

**Methods:**

We developed the training program based on a review of current academic HTA master’s and advanced programs globally, as well as an assessment of the training needs of the intended participants. The program consisted of five modules comprising 30 online sessions of four hours, with a mixture of lectures and work sessions complemented with self-study assignments and a targeted train-the-trainers program. We asked participants to report on their level of confidence in each learning objective at the start and the end of each module. A questionnaire was administered at the end of the program to evaluate participants’ satisfaction with study load, course content, and organization. This research was funded by USAID, reference number RFP-2021-11-18, through a contract with Management Sciences for Health under AID-121-C-17-00004. The contents do not necessarily reflect the views of USAID or the U.S. Government.

**Results:**

The five modules focused on the following: introduction to HTA; comparative effectiveness; health economics; qualitative evidence synthesis; patient and public involvement; and ethical, sociocultural, legal, and other relevant issues. Between 30 and 75 persons attended one or more of the training sessions. For each module, a learning effect was observed among participants. Among the 42 survey respondents, the average grade was excellent (9.5/10). The setup, organization, and content of the program and material provided, as well as the level of expertise provided by the teachers, were rated as very good.

**Conclusions:**

Participants agreed that the program was relevant and contributed to their professional growth. Twelve academics and staff from the HTA body were trained to help sustain the program in Ukraine. The project resulted in HTA training for doers, users, and trainers in Ukraine, and can be an example for other countries wishing to increase sustainable HTA capacity.

